# Peripheral immune cell profiles and tumor marker expression in high-risk HPV-infected cervical lesions: a comparative study

**DOI:** 10.3389/fonc.2026.1850475

**Published:** 2026-06-19

**Authors:** Hui-Ying Wang, Li-Rui Wu, Peng-Fei Guo, Lan-Peng Wang, Hong-Xia Li, Hong-Li Wang, Ya-Juan Wang, Shu-Juan Shao, Su-Ning Chen

**Affiliations:** 1 Department of Gynecology, Hebei PetroChina Central Hospital, Langfang, Hebei, China; 2 Langfang People’s Hospital, Langfang, Hebei, China

**Keywords:** cervical lesions, high-risk human papillomavirus, Ki-67, K-ras, regulatory T cells

## Abstract

**Objective:**

This study aimed to investigate the levels of peripheral blood immune cells CD4^+^, CD8^+^, CD56^+^, and regulatory T cells (Tregs) and the expression of tumor markers K-ras and Ki-67 in pathological tissues of patients with high-risk human papillomavirus (HR-HPV)-infected cervical lesions, and to explore their correlations with HPV-DNA viral load.

**Methods:**

A total of 240 female patients with HR-HPV infection treated at Hebei Central Hospital of Petroleum between January 2022 and December 2023 were retrospectively enrolled and categorized into three groups based on histopathological diagnosis: cervical cancer group (n=80), cervical intraepithelial neoplasia (CIN) group (n=80), and chronic cervicitis group (n=80). Peripheral blood mononuclear cells (PBMCs) were isolated, and the percentages of CD4^+^, CD8^+^, CD56^+^, and Tregs (defined as CD4^+^CD25^high^FoxP3^+^ lymphocytes) were determined by flow cytometry. Immunohistochemistry (IHC) was performed to assess the expression of K-ras and Ki-67 in cervical biopsy specimens. HPV-DNA viral load was quantified by fluorescent quantitative PCR. Correlation analyses were conducted between immune cell levels, tumor marker expression, and HPV-DNA content.

**Result:**

The cervical cancer group showed significantly lower CD4^+^ (31.45 ± 5.68%) and CD56^+^ (10.21 ± 2.15%) but higher CD8^+^ (29.84 ± 4.23%) and Tregs (8.98 ± 1.74%) than the CIN and cervicitis groups (all P<0.001). Proportions of K-ras^+^ (62.34 ± 10.57%) and Ki-67^+^ (68.45 ± 11.23%) cells were also highest in cervical cancer (P<0.001). HPV-DNA load increased progressively from cervicitis (median 58.16 RLU/CO) to CIN (103.83) to cervical cancer (173.68). Tregs (r=0.603) and CD8^+^ (r=0.628) correlated positively with HPV-DNA load, while CD4^+^ (r=-0.586) and CD56^+^ (r=-0.542) correlated negatively (all P<0.001). Both K-ras (r=0.647) and Ki-67 (r=0.689) showed positive correlations with HPV-DNA load (P<0.001).

**Conclusion:**

Patients with HR-HPV-infected cervical lesions exhibit distinct alterations in peripheral immune cell profiles and tumor marker expression. Assessment of these biomarkers may facilitate the early identification of cervical lesions associated with HR-HPV infection and inform the development of preventive and therapeutic strategies.

## 1 Introduction

Cervical cancer remains one of the most common malignancies affecting women worldwide, with its incidence and mortality rates continuing to rise globally (1, 2). The development of cervical cancer is strongly associated with human papillomavirus (HPV) infection, particularly infection with high-risk HPV (HR-HPV) types, which are recognized as the primary etiological agents in cervical carcinogenesis (3, 4). Although the introduction of prophylactic HPV vaccines has contributed to a reduction in cervical cancer incidence in certain regions, limited vaccination coverage and accessibility issues mean that cervical cancer continues to pose a significant public health burden in many countries (5). Consequently, the early detection and effective management of cervical lesions associated with HR-HPV infection remain urgent clinical priorities (6).

Accumulating evidence indicates that the host immune system plays a pivotal role in the pathogenesis of HPV infection and the progression of HPV-related cervical lesions (7, 8). HPV employs multiple immune evasion strategies, including downregulation of antigen presentation and modulation of the local immune microenvironment, to establish persistent infection and promote malignant transformation (9). Changes in the number and function of immune cell subsets—such as CD4^+^ helper T cells, CD8^+^ cytotoxic T lymphocytes, natural killer (NK) cells (CD56^+^), and regulatory T cells (Tregs)—have been closely linked to the progression of cervical lesions (10, 11). In parallel, the expression levels of tumor markers including K-ras and Ki-67 are also correlated with the severity and prognosis of cervical lesions (12, 13). Therefore, the systematic assessment of immune cell profiles and tumor marker expression may provide valuable information for the early diagnosis and management of cervical lesions associated with HR-HPV infection (14, 15).

The present study was designed to investigate the levels of peripheral blood CD4^+^, CD8^+^, CD56^+^, and Treg cells and the expression of K-ras and Ki-67 in pathological tissues of patients with HR-HPV-infected cervical lesions, and to explore their correlations with HPV-DNA viral load. A total of 240 patients with HR-HPV infection were enrolled and categorized into cervical cancer, CIN, and chronic cervicitis groups. Quantitative PCR, flow cytometry, and immunohistochemistry were systematically employed to characterize immune cell levels and tumor marker expression. This study aimed to elucidate the immune status and tumor marker expression profiles of patients with HR-HPV-related cervical lesions and to provide a scientific basis for early clinical diagnosis and treatment.

## 2 Materials and methods

### 2.1 Ethics statement

This study was conducted in accordance with the principles of the Declaration of Helsinki and was approved by the Institutional Review Board of Hebei Central Hospital of Petroleum (Approval No. HCH-IRB-2021-045). Written informed consent was obtained from all study participants prior to enrollment.

### 2.2 Study design and participants

This retrospective study included a total of 240 female patients with confirmed HR-HPV infection who were treated at the Department of Gynecology, Hebei Central Hospital of Petroleum, between January 2022 and December 2023. Based on histopathological diagnosis, patients were categorized into three groups: cervical cancer group (n=80), cervical intraepithelial neoplasia (CIN) group (n=80), and chronic cervicitis group (n=80). HR-HPV infection was confirmed by fluorescent quantitative PCR using cervical swab specimens. HPV genotyping was performed using a commercial HPV genotyping kit (Yaneng Bio, Shenzhen, China) capable of detecting 15 high-risk HPV types (HPV16, 18, 31, 33, 35, 39, 45, 51, 52, 53, 56, 58, 59, 66, and 68).

Inclusion criteria were as follows: (1) female patients aged 25 to 65 years; (this age range was selected because cervical cancer screening has been shown to have the greatest impact in this population, as supported by global screening guidelines and epidemiological data); (2) histopathologically confirmed diagnosis of cervical cancer, cervical intraepithelial neoplasia, or chronic cervicitis; (3) confirmation of HR-HPV infection by fluorescent quantitative PCR; (4) voluntary participation with signed informed consent; and (5) ability to cooperate throughout the study period without receiving other interventions that might affect the results.

Exclusion criteria were: (1) age younger than 25 years or older than 65 years; (2) presence of other malignancies or severe systemic diseases; (3) receipt of radiotherapy, chemotherapy, or immunotherapy prior to or during the study period; (4) severe liver or renal insufficiency or other conditions affecting immune system function; (5) mental illness or cognitive impairment precluding understanding and compliance with study requirements; and (6) pregnancy or lactation.

### 2.3 HPV-DNA detection

Cervical exfoliated cell samples were collected using a cervical brush. HPV-DNA extraction and fluorescent quantitative PCR were performed using a commercially available HR-HPV detection kit (Yaneng Bio, Shenzhen, China) following the manufacturer’s instructions. The PCR reaction was carried out on a LightCycler 480 II Real-Time PCR System (Roche Diagnostics, Basel, Switzerland). HPV-DNA viral load was expressed as copy number per 10,000 cells.

### 2.4 Peripheral blood sample collection and immune cell analysis by flow cytometry

Venous blood samples (3 mL) were collected from each patient upon admission, with collections performed consistently between 7:00 and 8:00 AM following an overnight fast to minimize circadian variation. Blood was drawn into EDTA-anticoagulated tubes and processed within 2 hours of collection. For flow cytometric analysis, peripheral blood mononuclear cells (PBMCs) were isolated by density gradient centrifugation using Ficoll-Paque PLUS (GE Healthcare, Chicago, IL, USA). Briefly, whole blood was diluted 1:1 with phosphate-buffered saline (PBS), layered carefully over Ficoll-Paque solution, and centrifuged at 400 × g for 30 minutes at room temperature without braking. The mononuclear cell layer (buffy coat) was aspirated, washed twice with PBS, and resuspended in RPMI 1640 medium. Cell viability was assessed by trypan blue exclusion; samples with viability <85% were excluded from analysis. Lymphocyte concentration was adjusted to approximately 5×10^6^ cells/mL. For surface marker staining, the following fluorochrome-conjugated antibodies (all from BD Biosciences, San Jose, CA, USA) were used: PE-Cy5-conjugated anti-CD4 (clone RPA-T4), FITC-conjugated anti-CD8 (clone HIT8a), and APC-conjugated anti-CD56 (clone B159). For Treg identification, a separate panel was used to define CD4^+^CD25^high^FoxP3^+^ lymphocytes. Briefly, cells were first stained with FITC-conjugated anti-CD4 (clone RPA-T4) and PE-conjugated anti-CD25 (clone M-A251) for 30 minutes at 4 °C in the dark. Following surface staining, cells were fixed, permeabilized using FoxP3 Fixation/Permeabilization buffer (eBioscience, San Diego, CA, USA), and then stained with APC-conjugated anti-FoxP3 (clone 236A/E7). After the final wash, cells were fixed with 0.1% paraformaldehyde and analyzed within 2 hours. Flow cytometric acquisition was performed on a FACSCanto II flow cytometer (BD Biosciences, San Jose, CA, USA) equipped with 488 nm and 633 nm lasers. For each sample, at least 50,000 events were acquired. Data analysis was performed using FlowJo software (version 10.8, BD Biosciences). Lymphocytes were first gated based on forward scatter (FSC) and side scatter (SSC) characteristics, and doublets were excluded. The percentages of CD4^+^, CD8^+^, and CD56^+^ cells were calculated as the proportion of positive cells within the total PBMC population. Tregs were identified as CD4^+^CD25^high^FoxP3^+^ lymphocytes and expressed as a percentage of CD4^+^ T cells.

### 2.5 Immunohistochemical staining for K-ras and Ki-67

Cervical biopsy or surgical tissue samples were obtained from all patients during diagnostic colposcopy-directed biopsy or surgical resection. Tissues were fixed in 10% neutral-buffered formalin for 24–48 hours, embedded in paraffin, and sectioned at 4-μm thickness. Paraffin sections were deparaffinized in xylene, rehydrated through a graded ethanol series, and rinsed in PBS.

For antigen retrieval, sections were placed in 10 mM sodium citrate buffer (pH 6.0) and heated in a pressure cooker for 15 minutes, followed by natural cooling to room temperature. Endogenous peroxidase activity was blocked by incubating sections in 3% hydrogen peroxide in methanol for 10 minutes at room temperature, followed by three PBS washes. Sections were then incubated with 5% normal goat serum for 30 minutes at room temperature to block non-specific binding. Primary antibodies against K-ras (rabbit monoclonal, clone 21, dilution 1:200; Proteintech Group, Rosemont, IL, USA) and Ki-67 (rabbit monoclonal, clone MIB-1, dilution 1:200; Proteintech Group, Rosemont, IL, USA) were added and sections were incubated overnight at 4 °C in a humidified chamber. The following day, sections were brought to room temperature for 1 hour, rinsed three times with PBS, and incubated with biotinylated goat anti-rabbit secondary antibody (dilution 1:500; Vector Laboratories, Burlingame, CA, USA) for 30 minutes at room temperature. Following additional PBS washes, sections were incubated with avidin-biotin-peroxidase complex (ABC kit, Vector Laboratories) for 30 minutes. Diaminobenzidine (DAB) substrate solution was applied for color development, with development time monitored under a light microscope (typically 2–5 minutes). Finally, sections were counterstained with hematoxylin, dehydrated through graded ethanol, cleared in xylene, and mounted with neutral mounting medium.

### 2.6 Evaluation of IHC staining

Immunohistochemical staining was evaluated independently by two experienced pathologists who were blinded to patients’ clinical information. For each tissue section, five randomly selected high-power fields (×400 magnification) were examined. In each field, at least 200 cells were counted, and the proportion of positively stained cells (nuclear staining for Ki-67; cytoplasmic and/or membranous staining for K-ras) was calculated. The mean percentage of positive cells from the five fields was used for statistical analysis.

### 2.7 Statistical analysis

Statistical analyses were performed using SPSS version 26.0 (IBM Corp., Armonk, NY, USA). Continuous variables were expressed as mean ± standard deviation (SD). Normality of data distribution was assessed using the Shapiro-Wilk test. For variables following a normal distribution, one-way analysis of variance (ANOVA) followed by Tukey’s *post hoc* test was employed for comparisons among the three groups. For variables not conforming to normal distribution (indicated in footnotes of tables), the Kruskal-Wallis H test followed by Mann-Whitney U tests with Bonferroni correction was used. Categorical variables were compared using the chi-square test or Fisher’s exact test, as appropriate. Correlations between immune cell levels, tumor marker expression, and HPV-DNA content were evaluated using Pearson’s correlation coefficient after confirming normality of the variables. All statistical tests were two-tailed, and a P-value < 0.05 was considered statistically significant. All statistical methods used for each table are specified in the corresponding table footnotes.

## 3 Results

### 3.1 Baseline characteristics

**Table 1** summarizes the baseline demographic and clinical characteristics of the three patient groups. No significant differences were observed among the groups with respect to age, body mass index (BMI), marital status, age at first sexual intercourse, pregnancy frequency, or delivery frequency (all P > 0.05). However, there was a significant difference in disease duration, with the cervical cancer group showing the longest disease course (3.48 ± 1.24 years), followed by the CIN group (2.35 ± 1.15 years), and the chronic cervicitis group having the shortest disease duration (1.98 ± 0.94 years; F = 27.582, P < 0.001).

**Table 1:** Baseline characteristics of study participants.

Characteristics	Cervical cancer group (n=80)	CIN group (n=80)	Chronic cervicitis group (n=80)	F/χ²	P
Age (years)	41.32 ± 7.85	39.87 ± 8.02	40.25 ± 8.13	0.659	0.519
Disease duration (years)	3.48 ± 1.24	2.35 ± 1.15	1.98 ± 0.94	27.582	<0.001
BMI(kg/m²)	23.45 ± 2.11	23. 12 ± 2.35	23.27 ± 2.28	0.315	0.730
Married, n (%)	62 (77.50%)	59 (73.75%)	60 (75.00%)	0.276	0.871
Age at first sexual intercourse (years)	20.45 ± 2.34	20.67 ± 2.21	20.78 ± 2.45	0.423	0.656
Pregnancy frequency (n)	2.85 ± 1.12	2.75 ± 1.34	2.80 ± 1.23	0.163	0.850
Delivery frequency (n)	1.90 ± 0.78	1.85 ± 0.82	1.88 ± 0.80	0.107	0.898

All continuous variables were analyzed by one-way ANOVA; categorical variables were analyzed by χ² test.

### 3.2 Levels of peripheral blood immune cells

**Table 2** presents the percentages of CD4^+^, CD8^+^, CD56^+^, and Treg cells in peripheral blood across the three groups. One-way ANOVA revealed significant differences among the three groups for all four immune cell parameters (all P < 0.001). *Post hoc* comparisons showed that the cervical cancer group had significantly lower percentages of CD4^+^ (31.45 ± 5.68%) and CD56^+^ cells (10.21 ± 2.15%) compared with the CIN and chronic cervicitis groups (both P < 0.001). Conversely, the percentages of CD8^+^ (29.84 ± 4.23%) and Treg cells (8.98 ± 1.74%) were significantly higher in the cervical cancer group than in the other two groups (both P < 0.001). The CIN group showed intermediate values between the cervical cancer and chronic cervicitis groups for all parameters.

**Table 2:** Comparison of peripheral blood immune cell percentages among the three groups.

Immune cell subset	Cervical cancer group (n=80)	CIN group (n=80)	Chronic cervicitis group (n=80)	F	P
CD4+ (%)	31.45 ± 5.68	38. 12 ± 6.21	40.34 ± 6.15	42.167	<0.001
CD8+ (%)	29.84 ± 4.23	22.67 ± 3.89	20.98 ± 4.02	75.302	<0.001
CD56+ (%)	10.21 ± 2.15	15.67 ± 2.87	17. 12 ± 3.04	85.467	<0.001
Tregs (% of CD4^+^ T cells)	8.98 ± 1.74	6.54 ± 1.82	5.89 ± 1.77	45.121	<0.001

All continuous variables were analyzed by one-way ANOVA; categorical variables were analyzed by χ² test.

### 3.3 Expression of K-ras and Ki-67 in cervical tissues

**Table 3** summarizes the expression levels of K-ras and Ki-67 in cervical tissue samples from the three groups. Significant differences were observed among the groups for both markers (P < 0.001). The proportions of K-ras-positive and Ki-67-positive cells were significantly higher in the cervical cancer group (62.34 ± 10.57% and 68.45 ± 11.23%, respectively) compared with the CIN group (45.23 ± 9.78% and 48.12 ± 9.23%, respectively) and the chronic cervicitis group (15.34 ± 5.67% and 17.56 ± 6.23%, respectively), with *post hoc* comparisons showing all pairwise differences were significant (P < 0.001).

**Table 3:** Comparison of tumor marker expression levels in cervical tissues among the three groups.

Tumor marker	Cervical cancer group (n=80)	CIN group (n=80)	Chronic cervicitis group (n=80)	F	P
K-ras-positive cells (%)	62.34 ± 10.57	45.23 ± 9.78	15.34 ± 5.67	212.514	<0.001
ki-67-positive cells (%)	68.45 ± 11.23	48. 12 ± 9.23	17.56 ± 6.23	239.683	<0.001

Data were analyzed by one-way ANOVA with Tukey’s *post hoc* test.

### 3.4 Comparison of HPV-DNA viral load among the three groups

HPV-DNA viral load was measured in all study participants. As shown in **Table 4**, there were significant differences in HPV-DNA load across the three groups. The cervical cancer group demonstrated the highest median HPV-DNA load [173.68 (IQR: 124.56–225.34) RLU/CO], followed by the CIN group [103.83 (IQR: 78.92–142.57) RLU/CO], and the chronic cervicitis group had the lowest median load [58.16 (IQR: 32.58–89.34) RLU/CO]. The Kruskal-Wallis test showed a significant overall difference (H = 58.324, P < 0.001), and *post hoc* Mann-Whitney tests with Bonferroni correction confirmed significant differences between all pairwise comparisons (all P < 0.01). These findings demonstrate that HPV-DNA viral load positively correlates with the severity of cervical lesions, consistent with previous studies.

**Table 4:** Comparison of HPV-DNA viral load among the three groups.

Group	n	Median HPV-DNA load (RLU/CO)	IQR	H	P
Chronic cervicitis	80	58.16	32.58–89.34	58.324	<0.001
CIN	80	103.83	78.92–142.57		
Cervical cancer	80	173.68	124.56–225.34		

Data were analyzed by Kruskal-Wallis H test followed by Mann-Whitney U tests with Bonferroni correction.

### 3.5 Correlations between immune cell levels and HPV-DNA content

**Figure 1:**
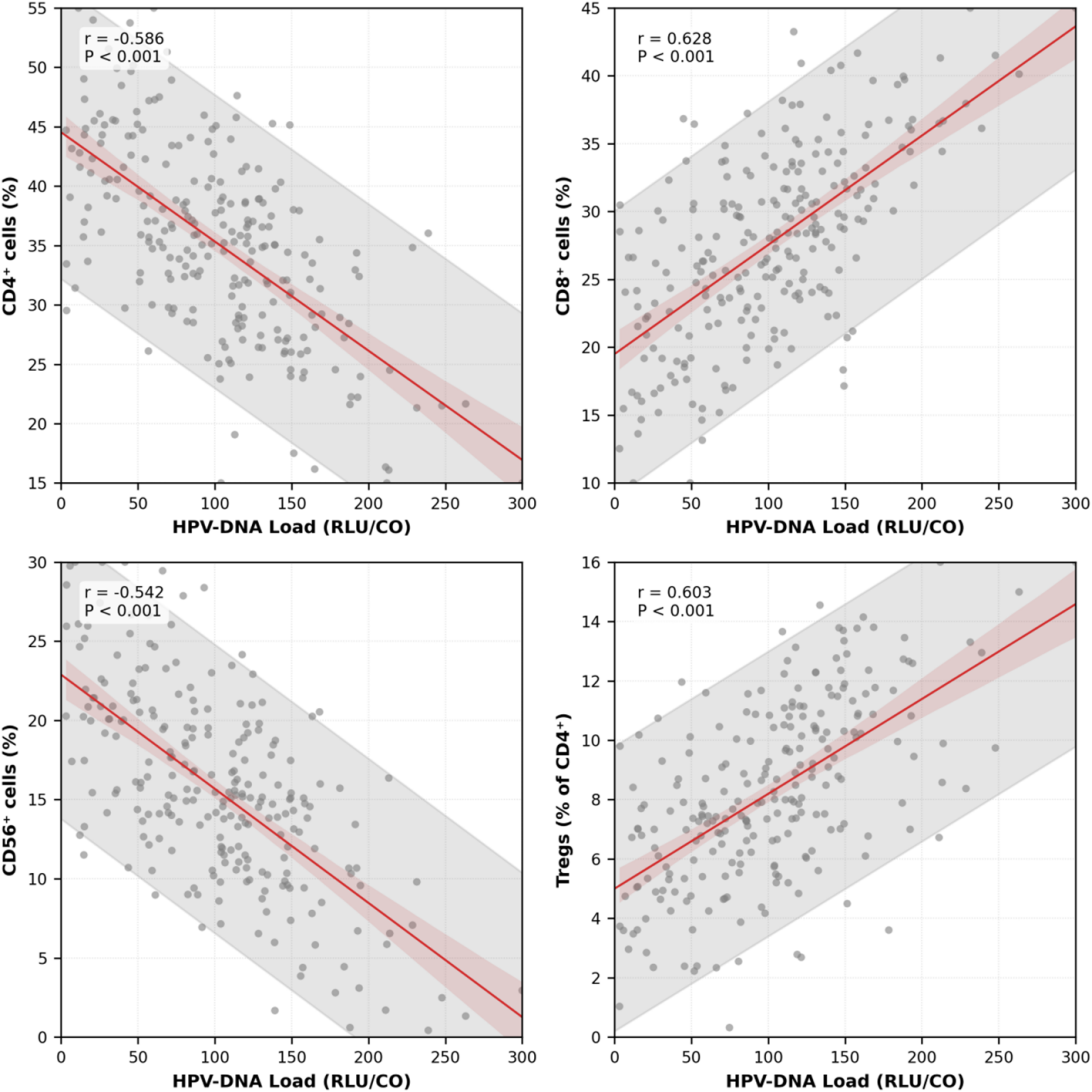
Scatter plots showing correlations between peripheral blood immune cell percentages and HPV-DNA content.

The correlations between peripheral blood immune cell percentages and HPV-DNA content are presented as scatter plots in **Figure 1**. Pearson’s correlation analysis revealed that Treg percentage (r = 0.603, 95% CI: 0.524 to 0.672, P < 0.001) and CD8^+^ cell percentage (r = 0.628, 95% CI: 0.552 to 0.695, P < 0.001) were significantly positively correlated with HPV-DNA content. In contrast, CD4^+^ cell percentage (r = -0.586, 95% CI: -0.658 to -0.503, P < 0.001) and CD56^+^ cell percentage (r = -0.542, 95% CI: -0.620 to -0.455, P < 0.001) showed significant negative correlations with HPV-DNA content.

The shaded area represents the 95% confidence band. n = 240 for all analyses.

### 3.6 Correlations between tumor marker expression and HPV-DNA content

**Figure 2:**
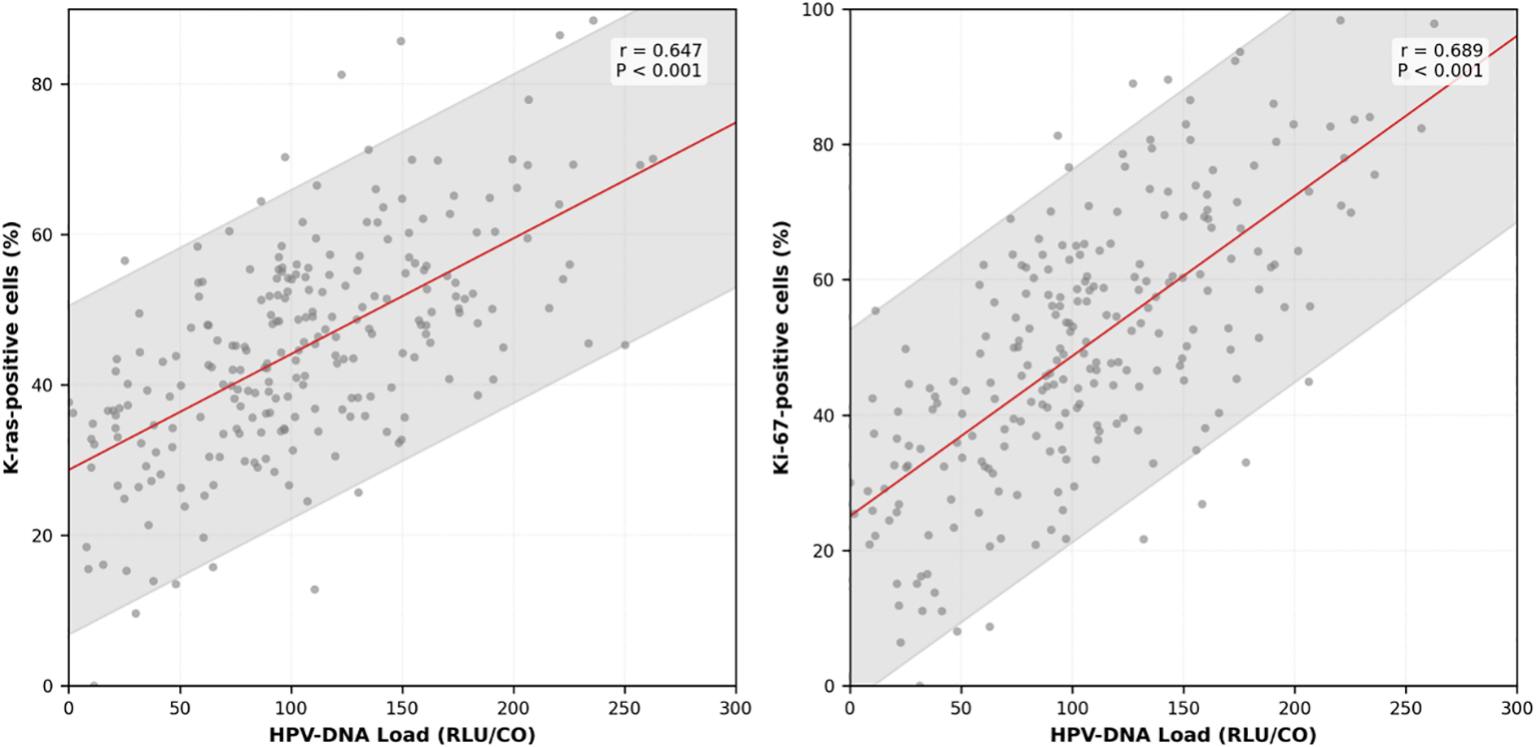
Scatter plots showing correlations between tumor marker expression levels and HPV-DNA content.

The correlations between K-ras and Ki-67 expression levels and HPV-DNA content are shown in **Figure 2**. Both K-ras-positive cell percentage (r = 0.647, 95% CI: 0.574 to 0.710, P < 0.001) and Ki-67-positive cell percentage (r = 0.689, 95% CI: 0.622 to 0.746, P < 0.001) were significantly positively correlated with HPV-DNA content.

The shaded area represents the 95% confidence band. n = 240 for all analyses.

## 4 Discussion

In this study, we systematically characterized the peripheral blood immune cell profiles and cervical tissue tumor marker expression patterns in patients with HR-HPV-infected cervical lesions across the disease spectrum from chronic cervicitis to CIN to invasive cervical cancer. Our main findings demonstrate that cervical cancer patients exhibit a distinct immunosuppressive phenotype characterized by reduced CD4^+^ helper T cells and CD56^+^ NK cells, along with increased CD8^+^ cytotoxic T cells and Tregs, coupled with elevated expression of K-ras and Ki-67. Moreover, these immune and tumor markers showed significant correlations with HPV-DNA viral load, suggesting their potential utility as indicators of disease severity and progression.

Cervical cancer remains a leading cause of cancer-related mortality among women globally, and HR-HPV infection is recognized as the necessary etiological factor for cervical carcinogenesis (2, 16) The host immune response plays a critical role in determining the outcome of HPV infection, with effective immune surveillance facilitating viral clearance and immune dysfunction contributing to viral persistence and malignant progression (17, 18). Our findings align with and extend previous observations by demonstrating that cervical cancer patients exhibit a significantly altered immune landscape characterized by decreased CD4^+^ and CD56^+^ cells and increased CD8^+^ and Tregs, with the magnitude of these changes correlating with HPV-DNA viral load (19, 20).

CD4^+^ helper T cells are essential for orchestrating effective anti-tumor immune responses, including the activation of cytotoxic CD8^+^ T cells and the induction of memory responses (21). The reduced CD4^+^ cell percentage observed in our cervical cancer group is consistent with the concept of progressive immune dysfunction during cervical carcinogenesis. HPV oncoproteins E6 and E7 are known to interfere with antigen presentation and promote the accumulation of immunosuppressive cells within the tumor microenvironment, leading to CD4^+^ T cell exhaustion (22, 23). This CD4^+^ reduction likely contributes to impaired clearance of HPV-infected cells, facilitating viral persistence and disease progression.

The elevated CD8^+^ cell percentage in cervical cancer patients, despite the concurrent increase in Tregs, appears paradoxical but may reflect an exhausted or dysfunctional CD8^+^ T cell population within the tumor microenvironment (24). In many solid tumors, including cervical cancer, tumor-infiltrating CD8^+^ T cells often exhibit markers of exhaustion such as PD-1, TIM-3, and LAG-3, rendering them ineffective despite their numerical presence (25). The strong positive correlation between CD8^+^ cell percentage and HPV-DNA content (r = 0.628) in our study supports the interpretation that higher viral loads are associated with greater CD8^+^ T cell recruitment, but likely with concomitant functional impairment.

Tregs are key mediators of immune tolerance and play a well-established role in suppressing anti-tumor immunity (26). The elevated Treg percentage in cervical cancer patients (8.98 ± 1.74%) and its positive correlation with HPV-DNA content (r = 0.603) are consistent with previous studies demonstrating Treg accumulation in HPV-related malignancies (27, 28). Tregs suppress effector T cell function through multiple mechanisms, including the secretion of immunosuppressive cytokines (IL-10, TGF-β), direct cell-contact inhibition, and metabolic disruption via CD39/CD73-mediated adenosine production (29). The increase in Tregs likely represents an HPV-mediated immune evasion strategy that facilitates viral persistence and promotes tumor progression.

CD56^+^ NK cells are critical components of the innate immune system, providing rapid responses against virus-infected and malignant cells without prior sensitization (30). The significantly reduced CD56^+^ cell percentage in cervical cancer patients (10.21 ± 2.15%) compared with CIN and chronic cervicitis patients, and its negative correlation with HPV-DNA content (r = -0.542), suggests that HPV infection may impair NK cell-mediated innate immunity. Mechanistically, HPV E6 and E7 proteins have been shown to downregulate NK cell-activating ligands on infected epithelial cells and to induce the secretion of immunosuppressive factors that inhibit NK cell activation and cytotoxicity (31).

Regarding tumor markers, Ki-67 is a well-established proliferation marker whose expression correlates with tumor aggressiveness and poor prognosis in various malignancies, including cervical cancer (32). The markedly elevated Ki-67-positive cell percentage in cervical cancer patients (68.45 ± 11.23%) and its strong positive correlation with HPV-DNA content (r = 0.689) align with the concept that HR-HPV integration and E6/E7 expression drive sustained cellular proliferation. Recent reviews have emphasized that a high Ki-67 labeling index correlates with advanced tumor stage, poor histologic differentiation, and reduced overall survival, underscoring its relevance in cervical cancer assessment (33).

K-ras is a member of the RAS family of small GTPases that transduces signals from membrane tyrosine kinase receptors to downstream protein kinase cascades regulating cell growth, differentiation, and survival (34). While K-ras mutations are more commonly associated with pancreatic, colorectal, and lung cancers, emerging evidence indicates that K-ras can cooperate with HPV E6/E7 in cervical carcinogenesis (35). Studies have shown that activation of ras promotes HPV-16 E6/E7 RNA expression and cyclin-dependent kinase activity, thereby contributing to the malignant transformation of HPV-immortalized keratinocytes (36). The elevated K-ras expression observed in our cervical cancer patients and its positive correlation with HPV-DNA content (r = 0.647) support the involvement of K-ras signaling in HPV-driven cervical cancer progression.

### 4.1 Comparison with previous studies

Our findings are consistent with and extend those of several recent studies. Voidazan (37) conducted a systematic review and meta-analysis on P16/Ki67 dual staining versus cytology for identifying high-grade CIN in HR-HPV-positive women, reporting superior diagnostic performance of dual staining compared to traditional methods. Their findings support the utility of combining proliferation markers with HPV testing in cervical lesion triage. A study by Li and colleagues (38) examining K-ras and C-myc expression in cervical cancer found that positive expression rates of K-ras were highest in cervical cancer, followed by CIN III, with no positive expression in chronic inflammation (P < 0.05), consistent with our K-ras findings. Moreover, their study showed that K-ras positive rate was significantly higher in poorly differentiated and advanced-stage cervical cancer tissues, suggesting K-ras expression is associated with higher malignancy and clinical stage. A systematic review on the cellular immune response to HR-HPV infection highlighted Tregs as a key cell type involved in cervical lesion progression, further validating our observations (39).

For Ki-67, our results are consistent with the meta-analysis by Piri and colleagues (40) demonstrating that Ki-67/MIB-1 has prognostic value for overall survival in cervical cancer patients, with a pooled HR of 1.63 (95% CI: 1.09-2.45; P < 0.05). Additional studies have confirmed the utility of p16/Ki-67 dual staining in predicting cervical lesion progression and enhancing cervical cancer screening accuracy (41, 42).

The strong correlations observed between immune cell profiles, tumor marker expression, and HPV-DNA content suggest that these biomarkers may have clinical utility in risk stratification and disease monitoring (43). The progressive changes in CD4^+^, CD8^+^, CD56^+^, and Tregs from chronic cervicitis through CIN to cervical cancer indicate that longitudinal monitoring of peripheral blood immune parameters could provide valuable information about disease trajectory. Similarly, assessment of Ki-67 and K-ras expression in cervical biopsy specimens may help identify patients at increased risk of progression from CIN to invasive cancer (44). The finding that all evaluated markers correlated with HPV-DNA viral load suggests that viral load measurement may serve as an integrating biomarker, with higher viral loads predicting more pronounced immune dysfunction and proliferative marker expression (45).

Recent advances in cervical cancer screening highlight the potential of biomarker-based approaches. For example, p16/Ki-67 dual staining has been shown to enhance the accuracy and effectiveness of cervical cancer screening programs, and combining HPV DNA load assessment with p16/Ki-67 staining can increase the sensitivity of CIN lesion diagnosis and predict outcomes (46, 47). Our findings suggest that incorporating peripheral blood immune cell analysis into such multimodal screening strategies might provide additional value.

### 4.2 Study limitations

This study has several limitations that should be acknowledged. First, the relatively modest sample size (n=80 per group) and single-center design may limit the generalizability of our findings. Multi-center studies with larger cohorts are needed to validate our observations.

Second, we did not include a healthy control group without HPV infection. This limits our ability to establish baseline reference values for peripheral blood immune cell percentages in healthy individuals, and to quantify the extent of immune dysregulation associated with HPV infection independent of cervical pathology. The absence of a normal control group precludes assessment of the magnitude of immunological changes relative to the general population.

Third, while this study evaluated correlations among immune cells, tumor markers, and HPV-DNA content, it did not prospectively assess the predictive or diagnostic value of combining these biomarkers. The sensitivity, specificity, and predictive accuracy of various biomarker combinations were not calculated, and no diagnostic algorithm was developed or validated. Future prospective studies are needed to establish whether combining peripheral blood immunophenotyping with tissue-based marker analysis improves early detection and risk stratification beyond conventional methods.

Fourth, our Treg definition using CD4^+^CD25highFoxP3^+^, while consistent with the traditional consensus for human Treg identification, did not include additional markers such as CD127 to exclude activated non-regulatory T cells. The consensus on an essential marker set for Treg analysis recommends at least CD3, CD4, CD25, CD127, and FoxP3 as the minimally required markers for defining human Treg cells. However, this study analyzed PBMCs that had been cryopreserved, and the permeability compromised CD127 stability, so FoxP3+ Tregs were gated using CD4^+^CD25highFoxP3^+^ strategy after excluding CD8^+^ cells, which may have been a limitation in precisely distinguishing Treg cells from activated effector T cells.

Fifth, we used the traditional histopathological classification (CIN) rather than the LSIL/HSIL terminology preferred in many recent publications. While CIN remains a valid histopathological classification system, and the selection criteria were histopathological biopsy confirmed, future studies may benefit from further sub-stratification of patients based on cytological HSIL/LSIL categories to investigate whether immune and tumor markers show differential patterns across these subcategories.

Sixth, this study did not evaluate the functional status of immune cells (e.g., cytokine production capacity, cytotoxic activity, or exhaustion marker expression) beyond cell enumeration. Future studies incorporating functional immune assays could provide deeper insights into the mechanisms of HPV-mediated immune evasion.

Finally, we did not perform long-term follow-up to assess clinical outcomes. Prospective longitudinal studies are required to determine whether baseline immune and tumor marker profiles predict disease progression, treatment response, or survival outcomes in patients with HR-HPV-infected cervical lesions.

## 5 Conclusion

In conclusion, this study demonstrates that patients with HR-HPV-infected cervical lesions exhibit a progressive pattern of peripheral blood immune cell dysregulation characterized by decreasing CD4^+^ helper T cells and CD56^+^ NK cells and increasing CD8^+^ T cells and Tregs as disease severity advances from chronic cervicitis through CIN to invasive cervical cancer. These changes are accompanied by elevated expression of the proliferation marker Ki-67 and the signaling molecule K-ras in cervical tissues, both of which correlate positively with HPV-DNA viral load. Our findings suggest that the combined assessment of peripheral immune cells and tissue tumor markers may contribute to the characterization of disease severity in HR-HPV-infected individuals. Future multi-center, prospective studies with extended follow-up are needed to validate these observations and to determine whether biomarker-based algorithms can improve clinical decision-making for patients with HR-HPV-related cervical lesions.

## Data Availability

The original contributions presented in the study are included in the article/supplementary material. Further inquiries can be directed to the corresponding author.
